# Development of a Federally Funded Demonstration Colorectal Cancer Screening Program

**Published:** 2008-03-15

**Authors:** Laura C Seeff, Amy DeGroff, Florence Tangka, Ena Wanliss, Anne Major, Marion Nadel, A Blythe Ryerson, Janet Royalty, Cynthia Gelb, Eddie Reed

**Affiliations:** Centers for Disease Control and Prevention, Division of Cancer Prevention and Control; Division of Cancer Prevention and Control, National Center for Chronic Disease Prevention and Health Promotion, Centers for Disease Control and Prevention, Atlanta, Georgia; Division of Cancer Prevention and Control, National Center for Chronic Disease Prevention and Health Promotion, Centers for Disease Control and Prevention, Atlanta, Georgia; Division of Cancer Prevention and Control, National Center for Chronic Disease Prevention and Health Promotion, Centers for Disease Control and Prevention, Atlanta, Georgia; Division of Cancer Prevention and Control, National Center for Chronic Disease Prevention and Health Promotion, Centers for Disease Control and Prevention, Atlanta, Georgia; Division of Cancer Prevention and Control, National Center for Chronic Disease Prevention and Health Promotion, Centers for Disease Control and Prevention, Atlanta, Georgia; Division of Cancer Prevention and Control, National Center for Chronic Disease Prevention and Health Promotion, Centers for Disease Control and Prevention, Atlanta, Georgia; Division of Cancer Prevention and Control, National Center for Chronic Disease Prevention and Health Promotion, Centers for Disease Control and Prevention, Atlanta, Georgia; Division of Cancer Prevention and Control, National Center for Chronic Disease Prevention and Health Promotion, Centers for Disease Control and Prevention, Atlanta, Georgia; Division of Cancer Prevention and Control, National Center for Chronic Disease Prevention and Health Promotion, Centers for Disease Control and Prevention, Atlanta, Georgia

## Abstract

Colorectal cancer is the second leading cause of cancer-related mortality among U.S. adults. In 2004, treatment costs for colorectal cancer were $8.4 billion.

There is substantial evidence that colorectal cancer incidence and mortality are reduced with regular screening. The natural history of this disease is also well described: most colorectal cancers develop slowly from preexisting polyps. This slow development provides an opportunity to intervene with screening tests, which can either prevent colorectal cancer through the removal of polyps or detect it at an early stage. However, much less is known about how best to implement an effective colorectal cancer screening program. Screening rates are low, and uninsured persons, low-income persons, and persons who have not visited a physician within a year are least likely to be screened.

Although the Centers for Disease Control and Prevention (CDC) has 15 years of experience supporting the National Breast and Cervical Cancer Early Detection Program for the underserved population, a similar national program for colorectal cancer is not in place. To explore the feasibility of implementing a national program for the underserved U.S. population and to learn which settings and which program models are most viable and cost-effective, CDC began a 3-year colorectal cancer screening demonstration program in 2005.

This article describes briefly this demonstration program and the process CDC used to design it and to select program sites. The multiple-methods evaluation now under way to assess the program's feasibility and describe key outcomes is also detailed. Evaluation results will be used to inform future activities related to organized screening for colorectal cancer.

## Introduction

Cancer is the second leading cause of death among U.S. adults, and colorectal cancer is the second leading cause of cancer-related deaths among U.S. men and women. In 2004, there were 145,083 new cases of colorectal cancer, and 53,580 people died of the disease (1). Although colorectal cancer affects both sexes and all races, men, African Americans, and Alaska Natives have disproportionately high incidences and mortality rates of colorectal cancer (2). In addition, colorectal cancer is expensive to treat: in 2004, the national cost of medical treatment for this disease was $8.4 billion (3).

Most colorectal cancers develop slowly from pre-existing polyps. This slow development provides an opportunity to intervene with screening tests, which can either prevent colorectal cancer through the removal of polyps or detect it at an early stage (4). Substantial scientific evidence shows that the incidence and mortality are reduced by regular screening (5-10). However, screening rates have been slow to increase (11), and it is not clear how best to implement an effective colorectal cancer screening program that reaches the people most in need. Uninsured persons, low-income persons, or those who have not seen a physician within the previous year are least likely to be screened (11).

The Centers for Disease Control and Prevention (CDC) has 15 years of experience supporting the National Breast and Cervical Cancer Early Detection Program (NBCCEDP) for the medically underserved population, but a similar national program for colorectal cancer is not in place (12). The NBCCEDP uses a comprehensive approach to breast and cervical cancer control, which includes providing early detection services, educational activities, public and private partnerships, and quality assurance measures. Clinically, screening for colorectal cancer is more complex than screening for either breast or cervical cancer because several screening tests are acceptable, each with a different recommended interval and each performed by different types of health care specialists. To understand better how to structure and implement population-level colorectal cancer screening and to explore which settings and program models are most viable and cost-effective, CDC began a 3-year colorectal cancer screening demonstration program in 2005. It was designed for low-income persons aged 50 to 64 years who are underinsured or uninsured for colorectal cancer screening.

This article describes briefly the process used to establish the CDC-funded Colorectal Cancer Screening Demonstration Program and the tools developed to implement this program. In accompanying reports in this issue of *Preventing Chronic Disease* (13-15), we present case-study and cost-study evaluation findings from the program start-up period, which we defined as beginning when the demonstration sites received their initial funds and ending when the screening itself began. Future reports will include evaluation results from the implementation phase of the program.

## Program Planning Process

In the summer of 2004, CDC held two meetings with 58 invited stakeholders, including clinicians and health scientists from CDC, other federal health agencies, partner organizations such as the American Cancer Society and Prevent Cancer Foundation, state health departments, and health systems such as managed care organizations. Also in attendance were representatives from Australia, the United Kingdom, and Italy, many with experience in organized colorectal cancer screening programs. During these stakeholder meetings, we used published data, the combined experience of meeting attendees, and consensus opinion to make key decisions that helped define the demonstration program. These decisions included the following:

Applicants for funds to implement a screening program could be any nonprofit medical entity that offered services to low-income persons who are underinsured for colorectal cancer screening.

When possible, screening programs would be flexible in their structure and design so they could meet the needs of the community they intended to serve.

Applicants would need to actively collaborate with their state's CDC-funded Comprehensive Cancer Control Program.

Because the U.S. Preventive Services Task Force (USPSTF) (16) recommends four screening tests with no one "best" test recommended, applicants proposing a screening program could choose which colorectal cancer screening tests to offer as long as

- The selected test or tests are recommended by USPSTF.
- The applicant has the capacity to offer the selected test or tests.

Providers within each selected program would be reimbursed for screening and diagnostic tests at the Medicare rate.

The focus of the screening programs would be on people aged 50 to 64 at average risk for colorectal cancer; younger persons would be eligible if they are at increased risk.

- Persons at increased risk for colorectal cancer because of a personal or family history of the disease would be eligible to receive screening services.
- Persons with colorectal cancer symptoms or at high risk of colorectal cancer because they already have inflammatory bowel disease or certain genetic conditions would not be eligible to receive services, because these conditions require frequent specialized care.

Programs would need to convene a local medical advisory board to address ongoing clinical issues.

As with the NBCCEDP, CDC funds would not be used for cancer treatment. To be eligible for consideration, applicants had to identify, in advance, sources that would provide treatment services for 1) cancers detected through the screening program and 2) complications that may arise during screening and diagnostic procedures.

## Site Selection and Program Features

Of the 39 applicants who competed for funds to start a demonstration project, 5 were selected and awarded a combined $2.1 million for year 1 of a 3-year program beginning in 2005. The successful applicants were the Maryland Department of Health and Mental Hygiene; Missouri Department of Health and Senior Services; Nebraska Department of Health and Human Services; Public Health – Seattle & King County, Washington; and Stony Brook University Medical Center, New York. The program's structure and the screening tests selected for the program are described in Figure 1.

**Figure.**
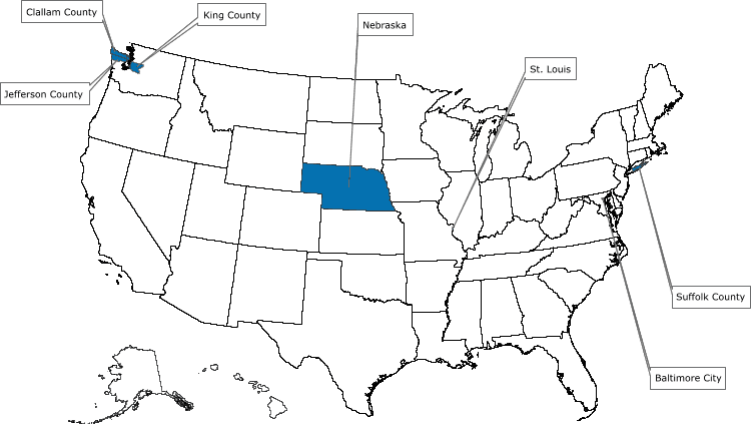
Colorectal Cancer Demonstration Screening Sites, 2005–2008

**Table:** 

Note: All programs provide screening and follow-up services to low-income persons aged 50–64 years, who are underinsured or uninsured for colorectal cancer. In addition, some programs focus on a specific demographic subgroup.

Each of the five programs is described in this issue of *Preventing Chronic Disease* (14). Program components in the five selected sites include clinical services (screening, surveillance, and diagnostic services), patient support, data collection and tracking, program management, public education and outreach, quality services measures, partnerships, and evaluation of program process and effectiveness. A multidisciplinary CDC team worked closely with the five selected sites during their start-up; the team included physicians, public health advisors, epidemiologists, program analysts, evaluators, health economists, data consultants, and health communications specialists.

## Materials Developed for the Colorectal Cancer Screening Program

During the start-up phase, which ranged from 9 to 11 months, CDC staff and program staff at each site collaborated to develop the program's materials and components. These include policies, a set of standard data elements (colorectal cancer clinical data elements or CCDEs), a data user's manual, data collection forms, a readiness checklist, a cost-assessment tool, and an evaluation plan. The cost-assessment tool and evaluation plan are described in another article in this issue of *Preventing Chronic Disease* (15). The readiness checklist, program policies, CCDEs, and data users' manual are available at http://www.cdc.gov/cancer/colorectal/what_is_cdc_doing/demonstration. Data collection forms were designed individually by each program site and are available on request.

### Program policies

CDC created a policy manual for this demonstration program. Each site also created its own individual policies, which usually mirrored the policies that CDC created for the overall program. Policies were developed on the following topics: 1) patient eligibility, 2) reimbursement, 3) reporting adverse events, 4) collecting and reporting data, and 5) medical advisory committees. A series of supporting documents to accompany the policy manual were developed by CDC in collaboration with all sites. These documents include guidelines for patient eligibility and ineligibility, reimbursable clinical services, data reporting requirements and schedules, and service quality indicators. These indicators were developed to capture data on the characteristics of the population being served by the program, the completeness of clinical follow-up and treatment, and the timeliness of clinical follow-up and treatment.

### Colorectal Cancer Clinical Data Elements

The CCDEs were developed by CDC, program sites, and two external clinical experts to ensure that information collected about clients' demographic characteristics, screening history, risk factors, screening and diagnostic tests, final diagnosis, and treatment would be consistent and complete. These data will be used to monitor the extent to which the five demonstration sites achieve the objectives of the demonstration project. They will also be used to guide future policy and program development. CDC provided technical assistance on data management and reporting to each of the five sites through routine communication and through tools that promote standard reporting, including a communication Web site for program participants, an extensive data dictionary, and software to validate the data set being submitted. Because of the short time frame and limited number of awardees participating in the program, we did not provide a standard data management system. 

### Quality assurance

Several measures were taken to emphasize the importance of delivering high-quality clinical services. Programs were made aware of important quality issues, including the frequent and inappropriate use of in-office rather than at-home fecal occult blood tests (FOBT) (17), wide variation in adenoma detection rates among endoscopists, and too-frequent surveillance following polyp detection (18). Teleconferences were conducted with CDC staff, endoscopists at all five sites, and a leading expert in assessing the quality of colorectal cancer screening about issues of quality and colonoscopy reporting guidelines. Programs and their participating endoscopists were encouraged to monitor their performance and follow the new standard colonoscopy reporting system (CO-RADS) (19), which specifies which elements should be included in colonoscopy reports, provides a standard method for reporting them, and specifies the key quality indicators that endoscopists should monitor periodically. The CCDEs include several factors that allow the quality of endoscopic services to be measured (e.g., whether the bowel preparation was adequate, whether the cecum was reached, whether polyps were completely removed).

### Public education materials

CDC's Screen for Life: National Colorectal Cancer Action Campaign (SFL) (http://www.cdc.gov/cancer/colorectal/sfl/) is a multimedia campaign that promotes colorectal cancer screening for adults aged 50 years or older. Programs were not required to use SFL materials, but materials that could be adapted for local use were developed by CDC staff and made available to the five programs. During start-up, two sites used the SFL materials and worked with CDC to adapt the SFL logo for their own program's use. Later, a third program elected to use SFL materials for patient education.

### Readiness checklist

CDC developed a readiness checklist (available at http://www.cdc.gov/cancer/colorectal/what_is_cdc_doing/demonstration) to be used during start-up to assist CDC and the five sites to monitor progress toward implementing the program. The items in the checklist corresponded to program components and include providing screening and diagnostic follow-up services, public education and outreach, data collection and tracking, patient support, partnership development and maintenance, quality assurance and professional development, and program management.

## Evaluation

A comprehensive evaluation of the colorectal cancer screening program is being conducted. It will provide information to guide our next steps. Evaluators engaged program and CDC stakeholders in developing a multisite and multiple-method evaluation to assess the program's implementation, outcomes, and efficiencies. Qualitative and quantitative evaluation approaches will be used 1) to analyze patient screening and diagnostic services, 2) to determine cost and cost-effectiveness, and 3) to conduct a longitudinal multiple-case study.

## State Programs

Colorectal cancer screening is rapidly becoming a priority at the local, state, and national levels. Several states (e.g., New York, Maryland) have long-standing programs; other states and cities (e.g., Colorado, New York City) recently began colorectal cancer screening; and others (e.g., Iowa, Wyoming*)* are planning to allocate new resources for organized colorectal cancer screening programs. CDC is working closely with the five demonstration program sites and communicating with other local and state partners who are taking steps toward establishing local or state programs so that lessons learned can be disseminated widely. Several pieces of legislation have been introduced into the U.S. Congress that, if passed, would establish a national colorectal cancer screening program.

Program activities for the demonstration sites will continue through August 2008. The evaluation findings published in this issue of *Preventing Chronic Disease* (13-15) and findings to be reported from the implementation phase of the demonstration program will be used to make adjustments during the demonstration period and to guide emerging programs and larger future efforts.
